# Heterologous Immunity between Adenoviruses and Hepatitis C Virus (HCV): Recombinant Adenovirus Vaccine Vectors Containing Antigens from Unrelated Pathogens Induce Cross-Reactive Immunity Against HCV Antigens

**DOI:** 10.3390/cells8050507

**Published:** 2019-05-26

**Authors:** Babita Agrawal, Nancy Gupta, Satish Vedi, Shakti Singh, Wen Li, Saurabh Garg, Jie Li, Rakesh Kumar

**Affiliations:** 1 Department of Surgery, Faculty of Medicine and Dentistry, University of Alberta, Edmonton, AB T6G2S2, Canada; Shakti1@ualberta.ca (S.S.); wl6@ualberta.ca (W.L.); Jiel@ualberta.ca (J.L.); 2 Department of Laboratory Medicine & Pathology, Faculty of Medicine and Dentistry, University of Alberta, Edmonton, AB T6G2S2, Canada; Gupta1@ualberta.ca (N.G.); vedi@ualberta.ca (S.V.); saurabh@ualberta.ca (S.G.); rakesh.rkumar1988@gmail.com (R.K.)

**Keywords:** Hepatitis C virus, heterologous immunity, adenoviruses, host response

## Abstract

Host immune responses play an important role in the outcome of infection with hepatitis C virus (HCV). They can lead to viral clearance and a positive outcome, or progression and severity of chronic disease. Extensive research in the past >25 years into understanding the immune responses against HCV have still resulted in many unanswered questions implicating a role for unknown factors and events. In our earlier studies, we made a surprising discovery that peptides derived from structural and non-structural proteins of HCV have substantial amino acid sequence homologies with various proteins of adenoviruses and that immunizing mice with a non-replicating, non-recombinant adenovirus vector leads to induction of a robust cross-reactive cellular and humoral response against various HCV antigens. In this work, we further demonstrate antibody cross-reactivity between Ad and HCV in vivo. We also extend this observation to show that recombinant adenoviruses containing antigens from unrelated pathogens also possess the ability to induce cross-reactive immune responses against HCV antigens along with the induction of transgene antigen-specific immunity. This cross-reactive immunity can (a) accommodate the making of dual-pathogen vaccines, (b) play an important role in the natural course of HCV infection and (c) provide a plausible answer to many unexplained questions regarding immunity to HCV.

## 1. Introduction

Hepatitis C virus (HCV) is a serious human pathogen and worldwide, ~170 million people are chronically infected with HCV [1]. Humans exposed to HCV can follow different trajectories: (a) exposed but not infected, (b) acutely infected and spontaneously cleared, (c) chronically/persistently infected and (d) chronically infected with spontaneous clearance [2,3]. It is believed that host immunity plays a prominent role in how these different outcomes arise in individuals exposed to HCV [4]. However, despite extensive research since the discovery of HCV in 1989, the exact mechanisms and events leading to each of the different courses of HCV infection remain elusive [5,6,7]. Understanding natural immunity against HCV is crucial for the development of prophylactic/therapeutic vaccines against HCV. Development of host immune responses against HCV has been extensively studied using mice, chimpanzees and human subjects. The role of cellular immunity in controlling HCV viremia has been unequivocally demonstrated, although the role of antibodies in protective immunity against HCV cannot be undermined [8].

These extensive studies have also led to many surprises, which are often sidelined in the quest to finding a definitive answer to the specific questions being asked. Besides the different courses disease can take after clinical HCV exposure, there are two confirmed yet unexplained observations worth noting: (1) there is a very high false positive diagnosis of HCV when using anti-HCV antibody in patient sera as a detection method, a technique that’s used in first-line diagnostic tests around the world, and (2) there are a number of reports describing that peptides derived from HCV antigens can specifically stimulate T cells enriched from PBMCs of unexposed and uninfected individuals [9,10,11,12,13,14,15,16,17,18].

In laboratory diagnosis of HCV, it is well documented that reliable diagnosis of HCV is not possible by only using HCV EIA diagnostic methods [12]. Among low risk immunocompetent individuals, the false positive results range from 15–60% even with using the third-generation diagnostic kits that detect the presence of antibodies against specific HCV antigens core, NS3, and NS5. A similar range of false positive diagnoses was found using first- and second-generation diagnostic kits that detect antibodies against NS4 or core, NS3 and NS4 as target antigens [9,11,13]. Such false positive results mandate a confirmatory HCV RNA test, leading to high costs and have a significant impact on the affected individuals [9,11]. These results suggest that either the assay methods are not specific or there are pre-existing antibodies present in humans that cross-react with HCV antigens.

A number of laboratories have performed extensive studies determining cellular immune responses against proteins and peptides derived from various HCV antigens, from HCV unexposed, acute clearing and chronically infected subjects [4]. While studies of T cell responses in acutely infected-clearing and chronically infected patients demonstrate a prominent role played by T cells in viral clearance, studies in unexposed and never infected, seronegative and aviremic, normal healthy humans provide a conundrum [15,17]. Earlier, it was suggested that they may represent occult infection, brief viral infection but without conversion to seropositivity, and loss of seropositivity following viral clearance [15,18,19]. However, it is still not clear how broad-epitopes reactive T cells are primed against HCV antigens in the absence of HCV infection.

Several earlier reports have tried to address these conundrums regarding HCV immunity, but without an adequate answer [15,18,19]. One plausible explanation for such a common presence of broad humoral and cellular immune components against HCV antigens in seronegative, aviremic and supposedly HCV-unexposed normal healthy individuals could be the occurrence of cross-reactive antibodies and T cells generated due to wide-spread infection with an unrelated pathogen, i.e., heterologous immunity [20,21]. Earlier studies reported T cells against an influenza virus epitope, which cross-react with an epitope from NS3 of HCV [22], however, a narrow one epitope specific cross-reactivity would not explain widely distributed cross-reactive antibody and T cell responses.

We have been studying recombinant adenovirus vectors (rAds) that express various HCV antigens as a way to understand the generation of protective immune responses against HCV antigens in both humans (ex vivo) and mice (in vivo) for vaccine purposes [23,24,25,26,27]. During these studies, we started to observe unusual cellular and humoral immunity against various HCV antigens even when we used replication-deficient, non-recombinant adenovirus vector (hu Ad5) not containing an HCV transgene, as controls for our recombinant Ads. After extensive studies, we confirmed robust amino acid homologies and immune cross-reactivity between various peptides from antigens derived from HCV and adenoviruses [28]. Subsequently, we published a review article summarizing these results in the context of other published literature and presented models with plausible mechanisms of how cross-reactive T cell and antibody responses might arise [29].

The identification of broad immune cross-reactivity between Ad and HCV has far-reaching implications on how we interpret the mechanisms involved in the various courses of HCV infection and ultimately, resistance to HCV infection. Further, there are also significant implications for vaccines being developed against a number of pathogens and their diseases where rAds are being used as vaccine candidates. The potential to generate accidental cross-reactive immunity against HCV might change the course of HCV infection/epidemiology in humans. In this article, we examined whether mice immunized with recombinant adenoviruses that contain transgene antigens from HCV or unrelated pathogens such as *Mycobacterium tuberculosis* (*Mtb*), human immunodeficiency virus (HIV) and Ebola virus (EBOV), can also induce cross-reactive humoral and cellular immunity against various HCV antigens. Using high-throughput screening methods, our results demonstrated that individual rAds containing HCV-NS3, *Mtb*-Ag85B, HIV-gag, HIV-nef and EBOV-ZGP antigens, all induce cross-reactive humoral and cellular immune responses in mice against HCV-derived core, NS3 and NS5 antigens, in addition to the transgene antigen- specific responses.

## 2. Methods and Materials

### 2.1. Adenovirus Vector

Replication incompetent human adenovirus 5 (Hu Ad5, denoted as Ad) without a transgene insert was amplified and titrated in human embryonic cell line 293A (HEK-293A) and transformed with an adenovirus E1 gene (QBiogene Inc., Carlsbad, CA, USA) to provide complementarity for virus production.

### 2.2. Cloning of Mtb Ag85B, HIV-Gag and HIV-nef into Plasmids

The Ag85B gene of *Mtb* (H37Ra) was PCR amplified from DNA isolated from *Mtb* bacteria using primers (Ag85B F primer: 5′-GAAGATCTATGACAGACGTGAGCCGAAAG-3′; Ag85B R primer: 5′-GAAGATCTCAGCCGGCGCCTAACGAACT-3′) and cloned into the commercial pCR 2.1 vector (Invitrogen Life Technologies, Thermo Fisher Scientific, Burlington, ON, Canada) to create pCR 2.1 Ag85B. Cloned fragments were verified by sequencing. The plasmid pCR 2.1 Ag85B was digested with BamHI, and the purified cDNA fragments were cloned into AdenoVator Transfer vector (pAdenoVator-CMV5-IRES-GFP; Qbiogene) generating CMV5/GFP/Ag85B.

The Nef and gag genes of HIV-1 were PCR amplified from the full-length clones of HIV-1 pNL4-3 kindly provided by Dr. Christopher Power at the University of Alberta. Primers used in this study for gag and Nef contain a *Bam*HI or Bgl II site (Gag F primer: 5′-GCGAAGATCTATGCCTATAGTGCAGAACCTCCAG-3′; Gag R primer: 5′-GCGAAGTACAAAACTCTTGCTTTATGGCC-3′; Nef F primer: CGGGATCCATGGGTGGCAAGTGGTCAAAAAGT-3′; and Nef R primer: 5′-CGGGATCCTCAGTTCTTGAAGTACTC-3′). The PCR products were cloned into the commercial pCR 2.1 vector (Invitrogen Life Technologies) to create pCR 2.1 gag and pCR 2.1 Nef. Cloned fragments were verified by sequencing. Both plasmids were digested with *Bam*HI or Bgl II, and the purified cDNA fragments were cloned into AdenoVator Transfer vector (pAdenoVator-CMV5-IRES-BFP; Qbiogene, Carlsbad, CA, USA) generating CMV5/BFP/gag and CMV5/BFP/nef.

### 2.3. Construction of Recombinant Adenovirus Vectors

pAdenoVator is a replication-deficient E1/E3 deletion mutant of adenovirus vector of the serotype 5 (Ad5) (QBiogene). pAdenoVator ΔE1/ΔE3DNA and the linearized DNA from transfer vectors CMV5/GFP/Ag85B, CMV5/BFP/Gag and CMV5/BFP/Nef were co-transformed in BJ5183-competent cells. Transfer and propagation in DH5α cells were used to select the positive recombinants. The Qiagen Plasmid Midi kit was used to purify recombinant DNA. rAd/Ag85B, rAd/gag and rAd/Nef were transfected in to 293A cells using transfection reagent Effectene (Qiagen, Valencia, CA, USA). Amplification and plaque assay were done in 293A cells for propagation and titration of the recombinant Ads, followed by storage in −80 °C.

Recombinant adenovirus (rAd) that expressed HCV antigen NS3 (rAd-NS3) were prepared and reported earlier by us [25,26].

An E1/E3-deleted Hu Ad5 that expressed ZEBOV glycoprotein (rAd-GoptZGP) was kindly provided by Dr. Gary Kobinger (University of Manitoba, Winnipeg, Canada). The recombinant viral vector was amplified in 293A cells [30] and quantified by using a standard plaque assay on 293A cells [30]. All of the Ad vectors (Ad, rAd-NS3, rAd-Ag85B, rAd-gag, rAd-Nef, and rAd-EBGP) were negative for the nucleic acid sequences of HCV antigens core and NS5 by PCR but positive for the respective transgene [28] (unpublished results).

### 2.4. Mice Immunizations

All animal experiments used in this study were approved by the University of Alberta’s Animal Care and Use Committee (ACUC) for Health Sciences and were conducted in accordance with the guidelines of the Canadian Council of Animal Care (CCAC). Six- to seven-week-old male or female C57Bl/6 mice were purchased from Charles River Laboratory (Charles River, Canada) and housed in a conventional animal facility (HSLAS) at the University of Alberta. Mice were immunized once or twice intramuscularly or intranasally (at 14-day intervals) using various doses (9 × 10^6^–2 × 10^7^ PFU/mouse): Ad (2 × 10^7^ PFU/mouse, twice, i.m.), rAd-NS3 (2 × 10^7^ PFU/mouse, twice, i.m.), rAd-Ag85B (2 × 10^7^ PFU/mouse, twice, i.m.), rAd-HIV Nef (2 × 10^7^ PFU/mouse, twice, i.n.), rAd-HIV gag (1 × 10^7^ PFU/mouse, twice, i.m.) or rAd-EBGP (9 × 10^6^ PFU/mouse, once, i.m.). Phosphate-buffered saline (PBS) immunized mice were used as a control. Specific details of dose and route of administration are also indicated in figures or figure legends. Mice were euthanized 8 days after the first or second immunization(s) and serum and spleen samples were collected.

### 2.5. Immunohistochemistry

Mice were immunized once intramuscularly with the (r)Ads, followed by euthanization and collection of quadriceps muscles at 12, 24 and 48 h. Sections of muscles (10 μm) were fixed on the slides and stained with anti-NS3, anti-core mAbs as follows. Two washes with 0.05% Tween phosphate-buffered saline (PBS) buffer for 2 min were followed by 0.2% Triton X-100 (0.2%) PBS cleaning. Incubation with 5% diluted normal goat serum (30 min) was used to block non-specific binding of the secondary Ab. Slides were incubated with Anti-CD16/32 mAb (1hr) followed by washing twice with 0.05% Tween PBS. Anti-core and anti-NS3 (Research Diagnostic Inc., Flanders, NJ, USA) primary antibodies were added at a 1:100 dilution subsequently (30 min), followed by two washes with 0.05% Tween PBS. Sections were incubated with 3% H_2_O_2_ with 0.1% sodium azide in 0.05% Tween PBS buffer (10 min) to deplete endogenous peroxidase activity. After two further washes, secondary antibody (biotinylated goat anti-mouse) was added to the slides. Subsequently, DAB (3,3′Diaminobenzidine) was added for 20 min followed by washing. As a last step, chromogen was added for 5 min, followed by washing twice. Drying and dehydrating with 95% and 100% ethanol and clearing with xylene was followed with mounting using mounting medium (Polysciences Inc., Niles, IL, USA).

### 2.6. Experimental Design for High Throughput Screening of Cross-Reactive Humoral and Cellular Immune Responses Induced by Recombinant Ads Containing Antigens from Unrelated Pathogens

**Figure 1:**
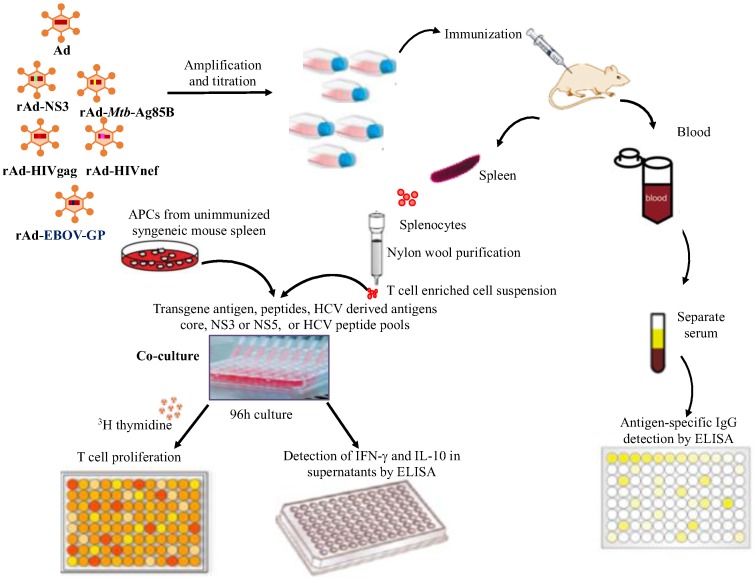
Schematic diagram depicting high throughput screening of cross-reactive humoral and cellular immune responses induced by recombinant Ads containing antigens from unrelated pathogens.

Although human subjects and clinical observations provide preliminary clues to the existence of cross-reactive immunity, immunizing mice with cross-reactive but unrelated pathogens provide the empirical test [31]. We, therefore, designed high throughput assays to determine cellular and humoral immune responses generated against both the transgene antigen and the core, NS3 and NS5 antigens of HCV (Figure 1).

We selected these three HCV antigens based on the extensive cross-reactivities we reported earlier [28]. Incorporation of ^3^H-thymidine into dividing cells was used to identify the proliferation of T cells, which also allows the testing of many antigens and peptides simultaneously in a high throughput (96-well) automated format. An automated cell harvester and β-counter, along with the appropriate controls, were implemented instead of highly labor-intensive flow cytometry for proliferation and intracellular cytokines against all antigens (proteins and peptides) tested. Further, the ^3^H-Tdr incorporation assay is more sensitive than colorimetric assays for detecting proliferation [32]. Combining the proliferation assay with ELISA assays for secreted cytokines IFN-γ and IL-10 also allowed us to determine the functionality of the proliferating T cells. Although, we designed these experiments in a high throughput manner, performing single cells assays using individual mice would be optimal. However, earlier, using single cell-based flow cytometry assays, we have confirmed the proliferation of CD4^+^ and CD8^+^ T cells and followed the cytokines produced by them [28]. We also determined the systemic induction of IgG in the sera of mice immunized against both the transgene and HCV antigens, by performing ELISA on serial dilutions (starting from 1:100 dilution) to eliminate possibility of a false random positive sample and avoid detection of natural antibodies [33] at high serum concentration of less than 1:100. In each assay, rhSOD and medium were used as negative controls. In each experiment, PBS immunized mice were used as controls. The recombinant HCV proteins (Genotype 1) core (c22-3), NS3 (c33c), NS5 (5.1.1) and control rhSOD were kindly provided by Chiron/Novartis and were analytical grade as used in the EIA diagnostic kits, to eliminate contamination or impurity issues. Peptide pools of HCV core, NS3 and NS5 antigens were prepared by mixing custom synthesized peptides as we reported earlier and are listed in Table 1 [28]. The transgene recombinant antigens or peptides were all highly pure and purchased from commercial sources. Details of the individual assays are provided as follows.

### 2.7. T Cell Proliferation Assay

Eight days after the last immunization, mice were euthanized, and spleens were collected. The spleens were pooled from all five mice and ground to single cell suspensions and filtered through a Falcon 100 μm nylon cell strainer. The cells were resuspended in 2 mL of medium and passed through an equilibrated nylon wool column. The column was washed after 45 min of incubation at 37 °C and the flow through contained the splenic T cells (1,2). These T cells were used in the experiments (~90% CD3^+^T cells). Proliferative responses were measured in triplicate cultures in 96-well flat-bottomed microtiter plates. A total of 4 × 10^5^ T cells from immunized mice and 4×10^5^ antigen resenting cells (splenocytes from unimmunized mice irradiated with 3000 rads) were incubated with different HCV-derived proteins (core = c22-3, NS3 = c33c; NS5 = NS5 SOD or control protein = rhSOD, kindly provided by Chiron/Novartis), synthetic HCV-derived peptide pools (Genscript Inc, Piscataway, NJ, USA) [28], recombinant HIV nef protein (Immune technology Inc., NY, USA), synthetic peptide from gag antigen (Genscript Inc.), sonicated *Mtb*(H37Ra) or synthetic *Mtb*-derived peptides of Ag85B (Genscript Inc.) at different concentrations as described in the figure legends. All peptides were custom synthesized by Genscript Inc., were >96% pure and are listed in Table 1. Plates were incubated for 4 days, and cells were pulsed with 0.5 μCi/well [^3^H]-thymidine (Amersham) for 12-18h and harvested on filter papers. The levels of [^3^H]-thymidine incorporated into the DNA of proliferating cells were counted in a Microbeta Trilux liquid scintillation counter (Perkin Elmer). Stimulation indices (SI) were calculated as CPM of antigen-stimulated culture/CPM of medium stimulated culture. The CPM in the medium stimulated cultures ranged from 150 to 1500 in different experiments. Data are represented as the mean stimulation indices ± SD (standard deviation) of triplicate cultures. SI values for HCV antigens, corrected for background from SI values in SOD-stimulated cultures (all < 2), are shown in the figures.

### 2.8. Cytokine ELISA

Levels of IFN-γ and IL-10 were assessed in culture supernatants collected from T cell proliferation assays using mouse IFN-γ and IL-10 ELISA kits following the manufacturer’s protocol (eBioscience, San Diego, CA, USA). A dilution of 1:2 to 1:10 was used for the samples with the standards ranging from 2 to 2000 or 4000 pg/mL. Plates were read and data were analyzed in FluoStar ELISA reader (BMG Labtech GmbH, Ortenberg, Germany). Cytokine concentration per mL of culture supernatant was determined by multiplying the calculated concentration with the dilution factor. The averages of these concentrations (pg/mL) from triplicate wells are shown.

### 2.9. Antibody ELISA

HCV antigen-specific cross-reactive and transgene-specific IgG antibodies were measured in sera collected from immunized mice using 96-well plates coated overnight (at 4 °C) with antigens from HCV (core, NS3 or NS5), HIV (nef), EBOV (gp) at 1 μg/mL or sonicated *Mtb* H37 Ra (1 × 10^6^ cfu/well) in 1× PBS. The next day, after blocking with 1% BSA at room temperature for 1 h, serial dilutions of serum samples (starting from 1:100) were added to the 96-well plate in 3 replicates and incubated at room temperature for 2 h. After application of serum, anti-mouse IgG labeled with alkaline phosphatase (AP) (Southern Biotech, Birmingham, AL, USA) was added and plates were incubated for 1 h. Color was developed by adding PNPP substrate (Southern Biotech). Plates were washed with 1× PBST (1× PBS with 0.1% Tween-20) after each incubation step. Absorbance was read using a FluoStar Optima ELISA Reader (BMG Labtech GmbH, Ortenberg, Germany), and OD values from HCV antigen coated plates, corrected for background from OD values in SOD coated plates, were plotted in the graphs shown here.

### 2.10. Statistical Analysis

Data were analyzed by Graphpad Prism software version 7.0 (Graphpad Software Inc., CA, USA). Data are presented as mean ± SD of triplicates and significant differences between groups were analyzed using Two-way ANOVA (Tukey’s test). *P*-values less than 0.05 were considered to be statistically significant and were denoted as *(*p* < 0.05), **(*p* < 0.01), ***(*p* < 0.001) and ****(*p* < 0.0001).

## 3. Results

### 3.1. In Vivo Immune Cross-Reactivity between Ad and HCV

**Figure 2:**
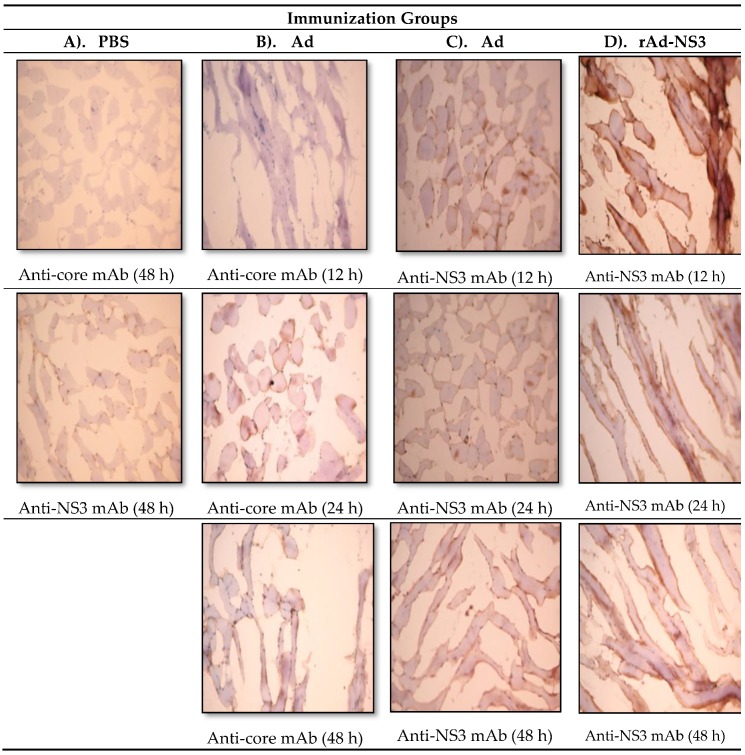
Cross-reactive binding of anti-core and anti-NS3 monoclonal antibodies to mouse quadricep muscles after a single immunization with adenoviral vector (Ad) or recombinant adenoviral vector (rAd-NS3). Male C57bl/6 mice (*n* = 5/group) were immunized once intramuscularly with (**A**) phosphate-buffered saline (PBS), (**B**) and (**C**) Ad, or (**D**) Ad-NS3 (2 × 10^7^ pfu/150 µL/mouse). Twelve, 24 or 48 h post immunization, quadricep muscles were removed and stained for hepatitis C virus (HCV) core and NS3 protein expression using the immunohistology procedure described in materials and methods.

Cross-reactive immune responses are usually demonstrated by using various ex vivo cellular and humoral immune assays, as we have also reported earlier [28]. To determine in vivo cross-reactivity between Ads and HCV, we immunized mice intramuscularly once in the quadriceps with Ad, rAd-NS3 or PBS, and observed thigh sections by immunohistology at 12, 24 and 48 h after immunization for binding by anti-HCV core or anti-HCV NS3 monoclonal antibodies (Figure 2). Both Ad and rAd-NS3 immunized mice demonstrated significant immune staining with commercially obtained monoclonal anti-NS3 and/or anti-core antibodies in thin sections taken at 12 h to 48 h post immunization. No immunostaining was observed in PBS immunized mice with anti-NS3 and anti-core antibodies. Also, isotype control antibodies did not show staining in Ad, rAd-core or rAd-NS3 immunized mice with (data not shown). The positive staining indicates the expression of cross-reactive antigens in the muscle after immunization with the non-replicating Ad vectors. These results provide direct in vivo evidence of immune (antibody) cross-reactivity between Ad and HCV antigens.

### 3.2. Induction of Cross-Reactive Humoral and Cellular Anti-HCV Immune Responses Induced upon Immunization with Recombinant Ad Vectors Individually Containing Antigens from HCV (NS3), Mtb (Ag85B), HIV (gag, Nef), and EBOV (GP)

**Figure 3:**
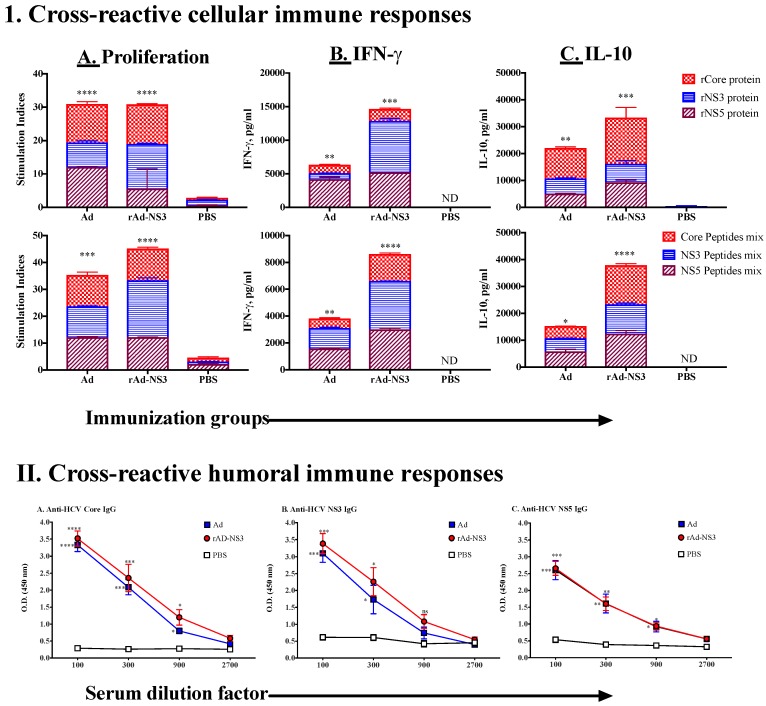
Intramuscular immunization with recombinant adenovirus expressing HCV antigen NS3 (rAd-NS3) induces cross-reactive immune responses against HCV core and NS5 antigens. Female C57bl/6 mice (*n* = 5/group) were immunized twice intramuscularly with Ad, rAd-NS3 (2 × 10^7^ pfu/150 µL/mouse), or PBS at 14-day intervals. Eight days after the second immunization, mice were euthanized and spleen and blood were collected. Cross-reactive cellular (I) and humoral immune (II) responses were measured against protein (HCV core, NS3, and NS5 at 5 μg/mL) or peptide pools (5 μg/mL) [core peptides #: 5, 14, 16, 17 & 27; NS3 peptides #: 2, 5, 6, 8 &10; NS5 peptides (NS5a peptides #: 6, 24; NS5b peptides #: 5, 19, 27), Table 1] [28]. [I]. Cellular immune responses upon stimulation with: proteins or peptides: (**A**) proliferation, (**B**) IFN-γ and (**C**) IL-10 production. [II]. Cross-reactive antibody response against HCV: (**A**) core, (**B**) NS3 and (**C**) NS5 antigens. All data represent mean + standard deviations of triplicate wells. *, **, *** and **** denote significant difference (*p* < 0.05, < 0.01, <0.001 and < 0.0001, respectively) between the experimental and PBS groups and ns represents not significant (*p* > 0.05). ND = not detectable.

In our earlier studies, we demonstrated that non-recombinant replication-deficient Ads induce cross-reactive immunity against HCV antigens [28]. To determine whether genetically expressing an HCV antigen (NS3) in the Ad vector would also lead to induction of cross-reactive immunity against various HCV antigens and/or further enhance immunity against NS3 of HCV, we immunized mice with non-recombinant Ad and recombinant Ad containing NS3 antigen (rAd-NS3) twice intramuscularly at the same dose (Figure 3). Although we have previously published data with Ad vector alone [28], we included it here again to use as a positive control group along with PBS immunized mice as negative controls in simultaneously performed experimental cohorts (Figure 3). The results demonstrated that immunization with both Ad and rAd-NS3 leads to induction of both cellular (proliferation and cytokines) and humoral immunity against all three antigens of HCV (core, NS3 and NS5) that we tested (Figure 3, I, II). All values were very significantly higher than the responses generated in PBS immunized mice. Proliferation and IFN-γ production appeared to be higher against NS3 antigen in rAd-NS3-immunized mice (Figure 3, I, A and B), however, cross-reactive T cell responses against core and NS5 were similar in both Ad and rAd-NS3-immunized groups. Similarly, antibody responses against core and NS5 were similar in both Ad- and rAd-NS3-immunized mice, whereas there was a slight increase in antibody response against NS3 in rAd-NS3-immunized mice compared to Ad immunized mice, but it was not significant. Interestingly, IL-10 responses were significantly increased in rAd-NS3-immunized mice against all three of the HCV antigens (both proteins and peptides, Figure 3, I, C) we tested, suggesting that including NS3 in the Ad vector in fact may be detrimental to the functionality of an induced cellular immune response and may negatively impact the beneficial effects of cross-reactive T cell immunity in terms of viral clearance.

**Figure 4:**
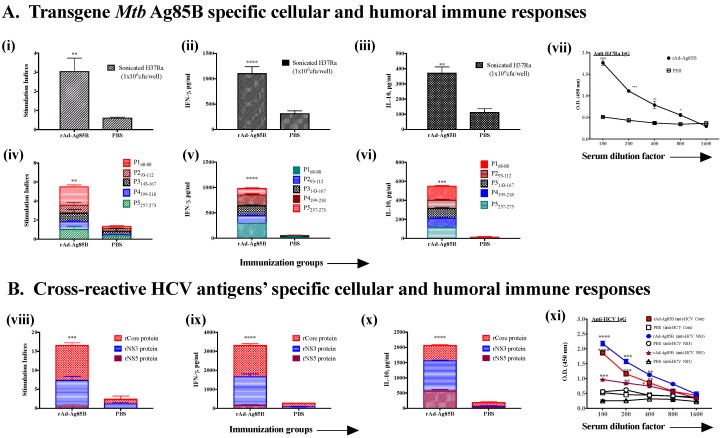
Intramuscular immunization with recombinant adenovirus expressing *Mtb* antigen Ag85B (rAd-Ag85B) induces cross-reactive immune responses to HCV-specific antigens as well as to *Mtb*-specific antigens. Male C57bl/6 mice (*n* = 5/group) were immunized twice intramuscularly with rAd-Ag85B (2 × 10^7^ pfu/150 µL/mouse) or PBS at 14 days interval. Eight days after second immunization, mice were euthanized and spleen and blood were collected. T cell and antibody responses were measured against both (**A**) *Mtb* antigen or peptides [sonicated *Mtb* (H37Ra 1 × 10^6^ cfu/well) or *Mtb* Ag85B synthetic peptides P1-P5 (Table 1) at 20μg/mL concentration] (i) proliferation, (ii) IFN-γ and (iii) IL-10, and peptides dependent (P1-P5) (iv) proliferation, (v) IFN-γ and (vi) IL-10 production, and (vii) anti-*Mtb* IgG antibody response in serum. (**B**). HCV antigens (core, NS3 and NS5 at 5 μg/mL). (viii) proliferation, (ix) IFN-γ and (x) IL-10 production and (xi) serum antibody responses. All data represent mean + standard deviations of triplicate wells. *, **, *** and **** denote significant difference (*p* < 0.05, < 0.01, < 0.001 and < 0.0001, respectively) between the experimental and PBS groups.

Mycobacterium tuberculosis (*Mtb*) is a significant human pathogen and worldwide efforts to generate a new vaccine against it are underway. Antigen 85B of *Mtb* is the major secretory protein in actively replicating *Mtb*, it is highly immunogenic and both humoral and cellular immune responses against it have been demonstrated in both latent and active-TB patients [34,35]. Ag 85B has been considered as a potential vaccine candidate against *Mtb* [36,37]. We immunized mice with recombinant Ad containing Ag85B of *Mtb* (rAd-Ag85B), and examined the cellular and humoral immune responses generated against *Mtb* (sonicated H37Ra used as antigen) and synthetic peptides derived from Ag85B (identified from the literature as associated with protective anti-*Mtb* immunity, [38] as well as HCV antigens core, NS3 and NS5 (Figure 4A,B). As expected, there was a significant T cell proliferation, cytokine and antibody responses generated against Ag85B in the mice immunized with rAd-Ag85B compared to PBS-immunized mice, which did not show these responses (Figure 4A). Interestingly, however, cross-reactive anti-HCV cellular and humoral responses were also generated, which were highly significant compared to PBS-immunized mice (Figure 4B).

**Figure 5:**
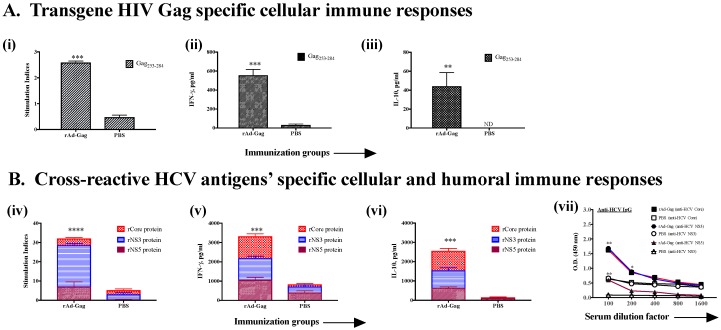
Intramuscular immunization with recombinant adenovirus expressing HIV Gag antigen (rAd-gag) induces cross-reactive HCV antigen-specific immune responses as well as gag-specific immune responses. Male C57bl/6 mice (*n* = 5/group) were immunized twice intramuscularly with rAd-Gag (1 × 10^7^ pfu/150 µL/mouse) or PBS at 14-day intervals. Eight days after the second immunization, mice were euthanized and spleen and blood were collected. T cell and antibody responses were measured against both (**A**) HIV [Gag antigen (Table 1) at 20μg/mL] and (**B**) HCV antigens (core, NS3, and NS5 at 5 μg/mL) (**A**). HIV-gag specific immune responses: Gag peptide-dependent (i) proliferation, (ii) IFN-γ and (iii) IL-10 production. (**B**). Cross-reactive immune responses against HCV antigens: (iv) proliferation, (v) IFN-γ and (vi) IL-10 production and (vii) serum antibody responses. All data represent mean + standard deviations of triplicate wells. *, **, *** and **** denote significant difference (*p* < 0.05, < 0.01, < 0.001 and < 0.0001, respectively) between the experimental and PBS groups. ND = Not detectable.

**Figure 6:**
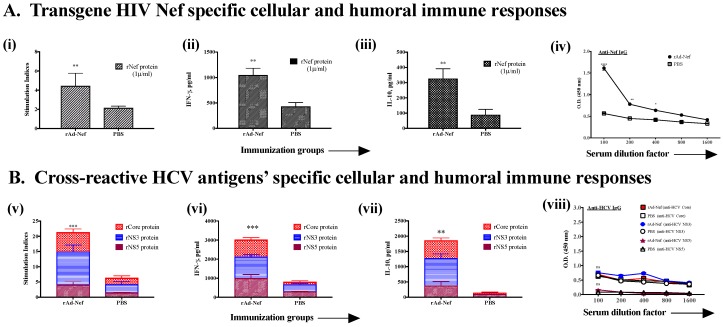
Intranasal immunization with recombinant adenovirus expressing HIV antigen Nef (rAd-Nef) induces cross-reactive HCV antigen-specific immune responses as well as Nef-specific immune responses. Male C57bl/6 mice (*n* = 5/group) were immunized twice intranasally with rAd-Nef (2 × 10^7^ pfu/30 µL/mouse) or PBS at 14-day intervals. Eight days after the second immunization, mice were euthanized and spleen and blood were collected. T cell and antibody responses were measured against both (**A**) HIV r-Nef protein (1 μg/mL) and (B) HCV antigens (core, NS3 and NS5 at 5 μg/mL). (**A**). Immune responses against Nef protein: (i) proliferation, (ii) IFN-γ and (iii) IL-10 production, and (vii) anti-Nef IgG antibody response in serum. (**B**). Cross-reactive immune responses against HCV antigens: (viii) proliferation, (ix) IFN-γ and (x) IL-10 production and (xi) serum antibody responses. All data represent mean + standard deviations of triplicate wells. *, **, *** and **** denote significant difference (*p* < 0.05, < 0.01, < 0.001 and < 0.0001, respectively) between the experimental and PBS groups and ns represents not significant (*p* > 0.05).

Human immunodeficiency virus (HIV), is another human pathogen, which urgently requires a prophylactic and/or therapeutic vaccine. Although phase III trials with a prophylactic HIV vaccine using the Ad vector were disappointing, immunotherapeutic vaccines containing T cell epitopes of Gag and Nef along with other antigens of HIV are currently being tested [39]. Further efforts are underway to understand the limitations of using the Ad vector for an HIV vaccine as well as ways to modify it [40,41]. In addition to i.m. injection, the mucosal route of immunization has been shown to be important for the success of an HIV vaccine due to its spread through both parenteral and mucosal routes [42]. We, therefore, examined rAd expressing Gag or Nef, by giving them intramuscularly and by intranasal routes to see if both routes induce systemic anti-HCV immune cross-reactivity (Figure 5 and Figure 6). Mice immunized with rAd-Gag led to significant T cell proliferation and cytokine responses against an immunodominant promiscuous peptide of Gag (Gag_253–284_) compared to PBS-immunized mice (Figure 5A). Antibody responses against Gag were not tested. Further, these mice demonstrated significant cellular and humoral cross-reactive immune responses against HCV antigens, compared to PBS-immunized mice (Figure 5B). Interestingly, intranasal immunizations with rAd-Nef (Figure 6), not only induced T cell proliferation, cytokines and systemic antibody responses against Nef (Figure 6A), it also induced systemic T cell proliferation and cytokine responses against various HCV antigens (Figure 6B). Interestingly, cross-reactive antibody responses were not significantly different than PBS-immunized mice (Figure 6B). The reasons for this are not clear, since an anti-Nef antibody response was generated to a significant degree. It is possible that cross-reactive humoral responses require a different dose of rAd when used by the intranasal immunization route, or Nef antigen of HIV, due to its immunomodulatory properties, modulates the generation of cross-reactive antibodies.

**Figure 7:**
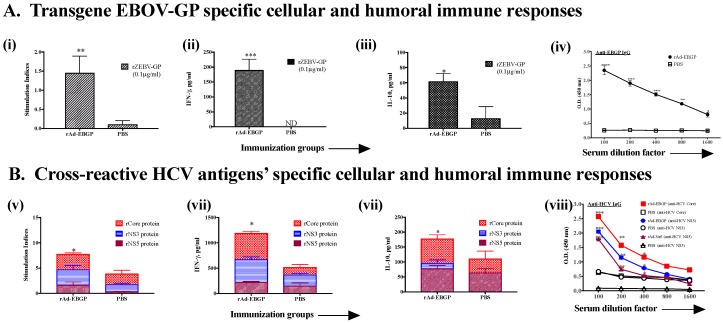
Intranasal immunization with recombinant adenovirus expressing Ebola virus (EBOV) glycoprotein (rAd-EBGP) induces cross-reactive HCV antigen-specific immune responses as well as transgene-specific immune responses. Male C57bl/6 mice (*n* = 5/group) were immunized once intramuscularly with rAd-EBGP (9.0 × 10^6^ pfu/150 µL/mouse) or PBS. Eight days after immunization, mice were euthanized and spleen and blood were collected. T cell and antibody responses were measured against both (**A**) EBOV-GP protein (0.1 μg/mL) and (**B**) HCV antigens (core, NS3 and NS5 at 5 μg/mL). (**A**). Immune responses against ZEBV glycoprotein: (i) proliferation, (ii) IFN-γ and (iii) IL-10 production, and (vi) anti-EBGP IgG antibody response in serum. (**B**). Cross-reactive immune responses upon stimulation with HCV antigens: (v) proliferation, (vi) IFN-γ and (vii) IL-10 production and (viii) serum antibody responses. All data represent mean + standard deviations of triplicate wells. *, **, *** and **** denote significant difference (*p* < 0.05, < 0.01, < 0.001 and < 0.0001, respectively) between the experimental and PBS groups.

Ebola virus (EBOV) is a member of the RNA virus family Filoviridae. EBOV outbreaks cause sporadic epidemic in certain parts of the world, however, due to high infectivity and mortality associated with EBOV, a public health emergency of international concern was declared by the WHO following the identification of EBOV in four countries in West Africa in the year 2014 [43]. Efforts to contain EBOV infection require a highly protective prophylactic vaccine. Hu Ad5 containing an enhanced antigenic expression cassette for the EBOV GP protein (Ad-GoptZGP) has been developed by Dr. Kobinger and was kindly provided to us [30,44]. A single intramuscular immunization of mice with Ad-GoptZGP at 9.0 × 10^6^ PFU/mouse led to modest T cell, but significant antibody responses against GP protein of EBOV (Figure 7A). Interestingly, this also led to significant induction of both cross-reactive cellular and humoral immune responses against HCV antigens core, NS3 and NS5, as compared to PBS-immunized mice (Figure 7B).

**Figure 8:**
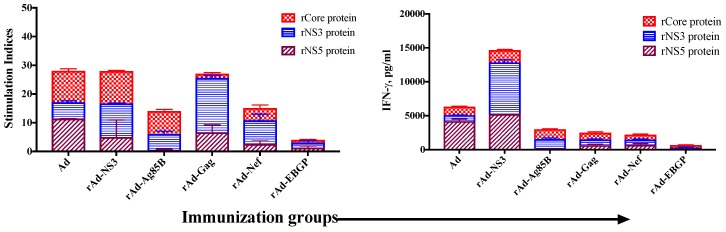
Cumulative summary of T cell proliferation and IFN-γ responses cross-reactive to HCV antigens as induced by Ad vector alone and various recombinant Ads. The T cell proliferation and IFN-γ responses generated against HCV antigens core, NS3 and NS5 upon immunization of mice with Ad vector alone, r-Ad-NS3, rAd-*Mtb* Ag85B, rAd-HIV Gag, rAd-HIV Nef and rAd-EBOV GP (from Figure 3, Figure 4, Figure 5, Figure 6 and Figure 7) are summarized for qualitative comparison.

Our results clearly demonstrate that recombinant Ads containing transgene antigens from diverse human pathogens are able to induce cross-reactive immunity against HCV antigens (Figure 3, Figure 4, Figure 5, Figure 6 and Figure 7). The T cell proliferation and IFN-γ responses induced by Ad and various recombinant Ads are summarized in Figure 8. Although Figure 8 suggests variations in the overall quantity of cross-reactive immunity induced, our assays only provide a qualitative measure. Also, this figure suggests that the nature of the transgene antigen may influence the level of heterologous immunity induced by Ads against HCV antigens. This would likely have clinical relevance in rAd-based vaccine approaches. More sophisticated single cells assays will be required to quantitively differentiate the cross-reactive immunity induced. Further, the data represent various rAd immunizations using different constructs of Ad (GFP or BFP containing CMV or Gopt expression cassette), routes (i.m. and i.n.), schedules (1× or 2×) and doses (9.0 × 10^6^ to 2 × 10^7^ pfu/mouse). Nevertheless, our data provide clear evidence of cross-reactive immunity induced by Ad and rAds against HCV antigens.

## 4. Discussion

Heterologous immunity is defined as immunity induced by one pathogen affecting immunity to another, unrelated pathogen, with varying beneficial or detrimental consequences on the course of infection with the second pathogen [31]. Concepts of heterologous immunity help explain many clinical observations and anomalies, especially the highly variable clinical course and outcome of viral infections in humans, which otherwise remain as mysteries [20,31]. Common respiratory pathogens such as influenza virus, coxsackie virus, adenovirus etc. continue to circulate among humans, often without serious consequences to human life, and thus provide a constant source of antigenic exposures [45]. Seroprevalence of Ad5 can approach >90% in certain areas and populations of the world, with the highest seropositivity in older populations [46]. Besides Ad5, the most common human adenovirus, >60 strains/types of Ads continually circulate in the human population, often without major health consequences [47]. Repeated exposure to natural Ad infections may result in subclinical persistent infections lasting for decades, and also may induce long-lived, cross-reactive humoral and cellular immune responses against various Ad antigens [48]. It has been shown that dendritic cells’ uptake of the immunocomplex of Ig-Ad can result in the conversion of DCs to tolerogenic DCs, allowing the induction of Tregs against Ads [49]. In addition to being a common respiratory pathogen, various strains of Ads from different host origins (humans, chimpanzees, bovine, canine etc.) are being tested as efficient vaccine and gene delivery vectors [48].

Our original observation that peptides derived from various HCV antigens possess strong homologies to various antigens of Ads and immunization of mice with Ads leads to robust heterologous immunity against HCV antigens, was unexpected [28]. We also carried sequence homology analyses between peptides derived from Ad5 and human immunodeficiency virus (HIV), West Nile virus (WNV), Influenza virus (IAV) and Dengue virus (DENV), and did not observe homologies at the same high levels as with HCV (Agrawal et al., Unpublished results). Whether low homologies (<30%) between viral antigens’ derived peptides can also result in cross-reactive immunity, needs to be investigated. In a subsequent review article [29], we elaborated the plausible molecular mechanisms that would explain this robust cross-reactivity between Ads and HCV. Concisely, the clonal selection theory suggests that one T cell clonotype exists for a single peptide epitope with the proper stringency in interaction and specificity. However, there is a vast imbalance in the number of peptides that an individual’s T cells have to respond to in a lifetime. There are only ~10^8^ T cell clonotypes available to respond to ~10^15^ peptide epitopes [50], suggesting that physiologically each T cell clonotype must respond to >10^6^ different peptide epitopes. At the molecular level, cross-reactivity of T cells can be explained by conformational plasticity of TCR-CDR loops, altered TCR:p-MHC docking geometry, structural degeneracy, molecular mimicry and/or flexibility of the peptide and MHC binding, or by a combination of any of these mechanisms. [29,51,52,53,54]. Based on these mechanisms in different combinations, even peptides with very little homology may have cross-reactive T cells recognizing them. Further, this potential variety makes it nearly impossible to predict cross-reactive T cell epitopes using in silico programs; identifying cross-reactive T cell epitopes is at present largely an empirical science. The literature shows that studies in mouse models can provide and validate information regarding cross-reactivity, as we have also shown [28,29,31]. Using one of the HCV core peptide epitopes (133–147), we presented a model of how a CD4^+^ and CD8^+^ T cell could cross-recognize an Ad5-derived peptide (in the context of MHC molecules) with only 53% homology (29). Similarly, antibodies can demonstrate this type of cross-reactivity in antigen recognition (29). Nevertheless, the observation of cross-reactive immunity between Ads and HCV is novel and opens new avenues of research in the field.

In this report we extend those observations and demonstrate that immunization of mice with various recombinant Ads, containing antigens from HCV, *Mtb*, HIV, and EBOV, all induce robust cross-reactive cellular and humoral immune responses against HCV antigens core, NS3 and NS5, in addition to responses generated against the intended transgene antigen (Figure 3, Figure 4, Figure 5, Figure 6, Figure 7 and Figure 8). In principle, these results from a mouse model provide means to study heterologous immunity in any vaccine design that uses rAd as a vector and its consequences in changing the course of HCV infection in humans. This is complicated, however, when we consider the multiple strains of Ads infecting a single individual multiple times in their life-time, and how cross-reactive immunity to HCV might affect the epidemiology and natural course of HCV infection. Further, the observation that multiple infection with Ads can generate regulatory T cells in humans [49], could help explain why there is high percentage of chronic HCV infections. It is possible that if T_regs_ are pre-selected by immunological tolerance/non-responsiveness to Ad antigens, they may also induce tolerance to cross-reactive HCV antigens. In our earlier studies with Ad-seropositive individuals, we observed that in both CD4 and CD8 T cells, IL-10 production was much more prevalent than IFN-γ production in responses to cross-reactive HCV antigens [28]. Although not conclusive, these preliminary results point toward modulation of HCV immunity because of earlier Ad exposure. The detailed criteria that account for beneficial vs. detrimental effects of heterologous immunity on HCV infection need to be investigated. However, it is likely that sub-optimal cross-reactive responses are in fact detrimental for a host exposed to HCV infection and at least partially account for high chronicity upon HCV infection. Unfortunately, in our small cohorts of human donors, none were seronegative for Ads, and we did not quantify the type of Ad-specific cellular immune responses, i.e., whether IFN-γ vs. IL-10 production against cross-reactive HCV antigens corresponds to a specific type of T cell memory or type of T_reg_ response present against Ad antigens.

Multiple aspects of HCV diagnosis, infection and progression of chronic disease may be affected due to cross-reactivity with adenoviruses, in both good and bad ways. Antibody-based HCV diagnostic tests have been reported to lead to an average of 35% false positive results among low risk individuals [12,29]. However, in contrast, specificity of the antibody-based diagnosis of HCV has been more broadly documented. Our results suggest that it is possible that false positive anti-HCV antibodies are due to seropositivity to Ad viruses. However, this idea requires further confirmation. It is also possible that the two natural courses of HCV disease are partly influenced by cross-reactivities induced through ubiquitous infection of humans with Ads (29). Further, multiple exposures to Ads may modulate cross-reactive T cells such that upon infection with HCV in the majority of individuals, efficient T cell immunity is not induced resulting in chronic infection (29). In addition, cross-reactive immune responses can explain certain unexplained observations associated with HCV immunity such as spontaneous re-activation of HCV-specific functional immune responses and viral clearance in some chronic patients, and the identification of HCV-specific memory T cells in HCV-naïve individuals [17,18,55,56,57,58,59,60,61]. It remains to be studied whether or how pre-existing immunity to adenoviruses shapes the course of natural infection with HCV in the human population and how they influence the attempts to develop vaccines against HCV.

The use of Ad vector for human vaccines presents a complex scenario. Exposure to common human Ads leads to induction of both neutralizing antibodies (nAbs) and T cell responses against Ad antigens. It has been suggested that the presence of nAbs can reduce the efficiency of a rAd-based vaccine. To overcome these limitations, different routes and doses of common human Ad vectors, rare human Ad types, or Ad vectors from different species altogether are being tested [48]. However, exposure of humans to common Ads induces T cells against antigens that are conserved in Ads from across subtypes and host-species, i.e., cross-reactive Ad-specific T cells are present in humans against rare human Ads as well chimpanzee Ads [61]. The functional attributes of these Ad-specific T cells could dictate the potential efficacy of rAd-based vaccine candidates in humans and also the nature of cross-reactive immunity against HCV. The impact of these complex factors/players on different natural courses of HCV infection and application of rAd-vectored vaccine as dual-pathogen vaccine warrants extensive study. In conclusion, recombinant adenovirus vectors containing various antigens from unrelated pathogens induce cross-reactive immunity against HCV. This is similar to the cross-reactive immunity to HCV seen in natural Ad infection. However, the use of rAd as dual pathogen vaccines in humans requires a better understanding of the complex interactions between the history of exposure to the pathogen and immunological responses to it in humans.

## Figures and Tables

**Table 1:** List of synthetic peptides used in T cell proliferation assays.

Pathogen	Protein	Peptide ^#^	Location	Amino Acid Sequence
HCV	Core	5	17–31	RRPQDVKFPGGGQIV
		14	53–67	SERSQPRGRRQPIPK
		16	61–75	RRQPIPKARRPEGRT
		17	65–79	IPKARRPEGRTWAQP
		27	105–119	PSWGPTDPRRRSRNL
	NS3	2	1367–1381	LSTTGEIPFYGKAIP
		5	1450–1464	SVIDCNTCVTQTVDF
		6	1127–1142	SSDLYLVTRHADVIP
		8	1467–1482	RRGRTGRGKPGIYRF
		10	1607–1622	MWKCLIRLKPTLHGP
	NS5a	6	2047–2066	VGPRTCRNMWSGTFPINAYT
		24	2317–2336	PPPRSPPVPPPRKKRTVVLT
	NS5b	5	2481–2500	DSHYQDVLKEVKAAASKVKA
		19	2691–2710	GENCGYRRCRASGVLTTSCG
		27	2811–2830	PLARAAWETARHTPVNSWLG
*Mtb*	Ag85B	P1	68–88	SGGNNSPAVYLLDGLRAQDDY
		P2	93–112	INTPAFEWYYQSGLSIVMPV
		P3	143–167	SELPQWLSANRAVKPTGSAAIGLSM
		P4	199–218	MGPSLIGLAMGDAGGYKAAD
		P5	257–273	NELGGANIPAEFLENFV
HIV	Gag	Gag_253-284_	253–284	NPPIPVGEIYKRWIILGLNKIVRMYSPTSILD

Peptide ^#^. Codes of peptides used throughout the manuscript.
